# An Excellent Appointment

**Published:** 1897-03

**Authors:** 


### AN EXCELLENT APPOINTMENT.

Governor Clough, of Minnesota, recently appointed Dr. M. H. Reynolds, of St. Paul, a member of the State Board of Health of the "Gopher State." This recognition of a veterinarian upon the State Board of Health of that State is certainly a step in the right direction and a very commendable one in every respect. Further, it is in keeping with the advanced action of the State of Minnesota and with the measure adopted by the city of St. Paul providing for an inspection of its milk-supply and an examination of the herds outside of the city, the milk products of which are offered for sale within the corporate limits. In the failure to reappoint Dr. Hewitt, long a member of the board, there can be no reflection cast upon the appointing power on the grounds of his failure to select one well equipped for the position. Dr. Reynolds is an earnest student, a graduate in human and comparative medicine, a skilled teacher, an advanced veterinarian, and one already selected by the State to take charge of a very responsible educational position in a State institution of learning. There can only be the greatest good to the largest number of people of the commonwealth through this recognition of Dr. Reynolds. The board's efficiency and its work will be broader and more far reaching, and its results more valuable in every way, not alone to that State, but to the whole country, as step by step it shall adopt measures for the better control and solution of the many questions involved in a better and more wholesome food-supply as well as the control of those diseases that are closely related to man and the lower animals.

We trust that the example set by Governor Clough will be followed in many other States by similar recognition of members of the veterinary profession, many of whom are particularly well equipped to fill positions of this kind.

